# Assessing fatty acid-induced lipotoxicity and its therapeutic potential in glioblastoma using stimulated Raman microscopy

**DOI:** 10.1038/s41598-021-86789-9

**Published:** 2021-04-01

**Authors:** Yuhao Yuan, Niraj Shah, Mohammad I. Almohaisin, Soumit Saha, Fake Lu

**Affiliations:** 1 Department of Biomedical Engineering, Binghamton University, State University of New York, Binghamton, NY 13902 USA; 2 Department of Psychology, Binghamton University, State University of New York, Binghamton, NY 13902 USA

**Keywords:** CNS cancer, Multiphoton microscopy

## Abstract

Glioblastoma multiforme (GBM) is the most aggressive primary brain tumor. The effectiveness of traditional therapies for GBM is limited and therefore new therapies are highly desired. Previous studies show that lipid metabolism reprogramming may be a potential therapeutic target in GBM. This study aims to evaluate the therapeutic potential of free fatty acid-induced lipotoxicity for the suppression of glioma growth. U87 glioma cells are treated with three fatty acids (FAs): palmitic acid (PA), oleic acid (OA), and eicosapentaenoic acid (EPA). Uptake of the FAs and formation of lipid droplets (LDs) are imaged and quantified using a lab-built stimulated Raman scattering (SRS) microscope. Our results show that a supply of 200 µM PA, OA, and EPA leads to efficient LDs accumulation in glioma cells. We find that inhibition of triglycerides (TAGs) synthesis depletes LDs and enhances lipotoxicity, which is evidenced by the reduced cell proliferation rates. In particular, our results suggest that EPA treatment combined with depletion of LDs significantly reduces the survival rate of glioma cells by more than 50%, indicating the therapeutic potential of this approach. Future work will focus on understanding the metabolic mechanism of EPA-induced lipotoxicity to further enhance its anticancer effects.

## Introduction

Glioblastoma multiforme (GBM) is a fast-growing aggressive brain tumor. The major therapies for GBM treatment include surgery, radiotherapy, and chemotherapy. Complete surgical resection of the tumor is challenging due to the infiltrative nature of GBM. Temozolomide (TMZ) is the primary chemotherapeutic drug^1^. However, GBM is widely reported with resistance to TMZ leading to a high recurrence rate and poor prognosis outcomes^2–4^. Drug resistance in GBM is also prominent with more recent anti-angiogenic agents and immunotherapeutic treatments^5^. To find new treatments for GBM, a deeper understanding of GBM pathophysiology is needed. Previous studies have shown that glioma cells exhibit abnormal lipid metabolism, which plays an important role in intensifying glioma aggressiveness^6,7^.

Unusual accumulation of lipid droplets (LDs) was found in both cultured glioma cells and surgically removed fresh tissue from glioma patients^8^. LDs have long been considered as a simple reservoir of neutral lipids, mostly triglycerides (TAGs) and cholesteryl esters (CEs). Recently more and more essential functions of LDs have been discovered, which are associated with cell cycle, cell proliferation, migration, adaptive drug resistance, and signaling^9–13^. It is also reported that LDs distributed abnormally in glioma tissue and LDs deposition increased in apoptosis or necrosis tissue in GBM^14,15^. Increasing evidence suggests that LDs may be a potential biomarker and metabolic target for GBM treatment^6^.

**Figure 1:**
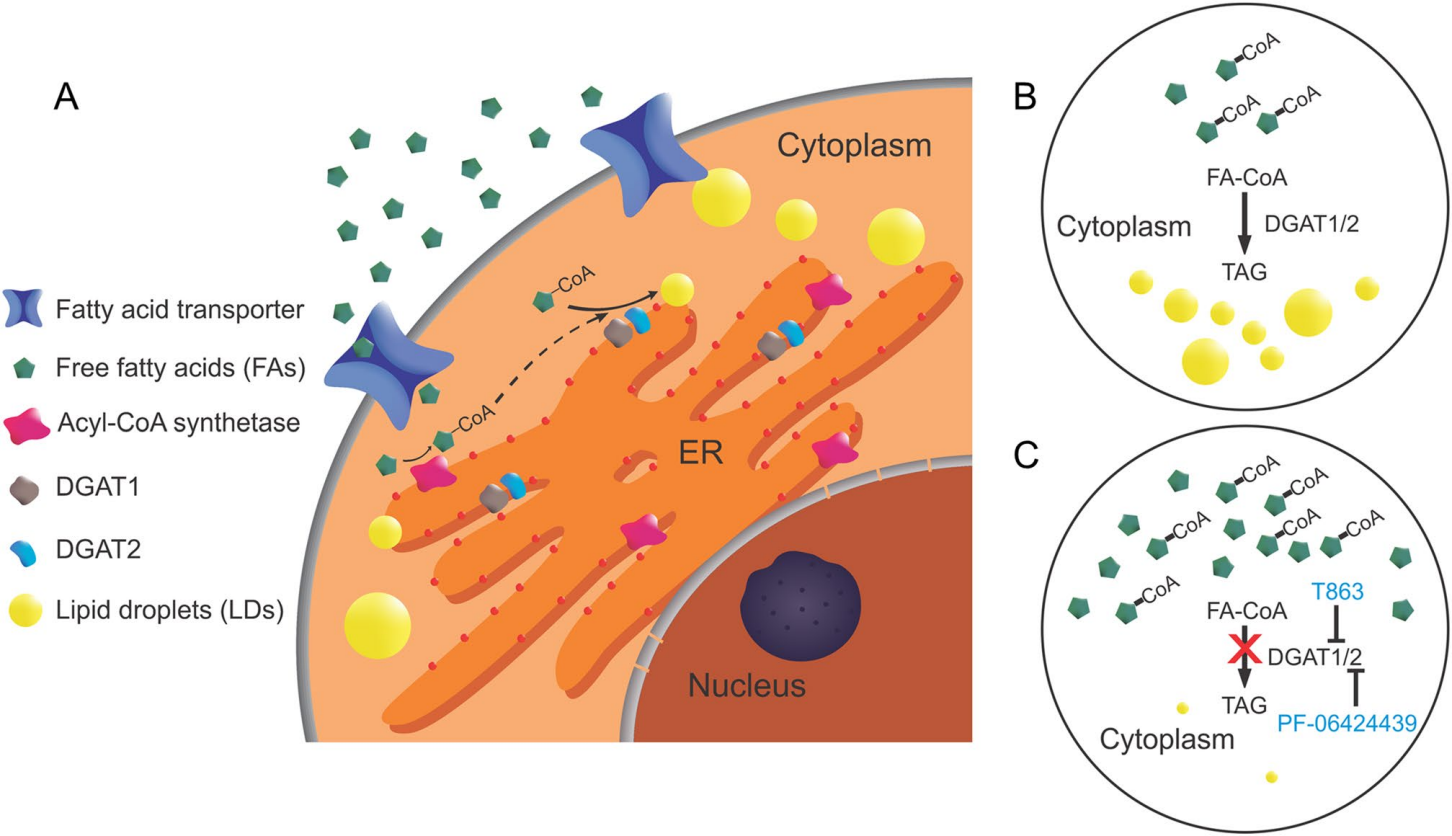
(**A**) Illustration of TAG synthesis and lipid droplets (LDs) formation from free fatty acids (FAs). Free FAs enter the cells through passive diffusion and fatty acid transporters and are activated into the form of acyl-CoA with the help of acyl-CoA synthetase. After further modification by related enzymes, acyl-CoA eventually forms TAG and LDs in the presence of DGAT1 and DGAT2. (**B**) The adipogenesis pathway shows the last step of lipogenesis involving enzymes DGAT1/2. TAG synthesis and LD formation are normal when DGAT1/2 are functioning properly. (**C**) Inhibition of DGAT1 and DGAT 2 using small-molecule inhibitors T863 and PF-06424439, respectively, to deplete cellular LDs. When DGAT1/2 are inhibited, TAG synthesis and LD formation are reduced, leading to increased free FAs. *ER* endoplasmic reticulum, *DGAT* diglyceride acyltransferase.

Adipogenesis for LDs biosynthesis involves multiple synthetases as briefly illustrated in Fig. 1. Uptake of extracellular free fatty acids (FAs) by glioma cells relies on both passive diffusion and fatty acid transporters^16–19^. With the help of acyl-CoA synthetase, FA is converted to FA-CoA as an active form for cellular catabolism and anabolism^20^. The overmuch FA-CoAs will eventually form TAGs catalyzed by the diglyceride acyltransferases (DGAT1 and DGAT2) and will be stored in LDs^11,21^.

Glioma cells exhibit altered lipid metabolic activities, including elevated uptake of free FAs or de novo lipogenesis. Under abnormal physiological conditions, such as hypoxia in the tumor microenvironment or extra supply of free FAs, more LDs will accumulate in the cytoplasm of cells^13^. It is known that the process of adipogenesis converts cytotoxic free FAs into non-toxic neutral lipids, which provides a protective mechanism for cells to relieve lipotoxicity induced by excess free FAs^14^. Lipotoxicity has been observed in non-adipose tissue and various human diseases^22^. Hereby, we hypothesize that via inhibition of TAGs synthesis and LDs formation, free fatty acid-induced lipotoxicity may be used as a new approach to suppress glioma cell growth and proliferation.

FAs are the direct substrates for TAG biosynthesis. Based on the degree of unsaturation (i.e., the number of double bonds), FAs could be classified into saturated fatty acids (SFAs), monounsaturated fatty acids (MUFAs), and polyunsaturated fatty acids (PUFAs). In GBM tissue, the most abundant two FAs are palmitic acid (PA; 16:0) and oleic acid (OA; 18:1)^23^. It is also reported that ω3-PUFAs, such as eicosapentaenoic acid (EPA; 20:5), induced cell death and increased radiotherapy responsiveness of glioma cells^24,25^. Based on these facts, we designed our study to test the three representative FAs, which are highly related to glioma cells with different saturation degrees: PA, OA, and EPA to understand their roles in lipotoxicity, LDs formation, and cancer cell growth suppression.

Stimulated Raman scattering (SRS) microscopy is an emerging technology for label-free chemical imaging of biomolecules including lipids, proteins, and nucleic acids^26–31^. SRS is a multiphoton process using two coherent ultrafast laser beams to excite the native sample. Due to the stimulated Raman signal amplification, SRS imaging is faster than Raman mapping by a few orders of magnitude, achieving imaging speed similar to confocal and fluorescence microscopy^32^. In addition, spectral SRS imaging has been used to analyze the major chemical composition of LDs^33,34^. The feasibility and great potential of SRS imaging of LDs in live cells have been demonstrated^30^. SRS chemical imaging is quantitative because the SRS signal intensity is proportional to the concentration of the targeted chemical bonds for imaging^35^.

In this study, we treated glioma U87 cells with PA, OA, and EPA, separately, at different concentrations. We observed and quantified LDs formation and changes of unsaturation level of LD lipids with label-free SRS imaging. We found that PA or EPA treatment reduced glioma cell viability, but OA treatment enhanced cell viability and proliferation, which were consistent with the previous studies^23,36,37^. By inhibiting the final step of the biosynthesis of TAG (i.e., inhibiting DGAT1 and DGAT2) for LDs formation^38–41^, the LDs were significantly reduced with all three FAs treatments. We also found that complete depletion of LDs reduced the cell survival rate of glioma cells treated with OA or EPA, but rescued PA-treated cells to some extent. The long-chain polyunsaturated EPA showed the highest lipotoxicity in suppressing glioma cells. These results reveal the complicity of lipotoxicity induced by various types of free FAs and lipids. By selecting proper FAs and by inhibiting LDs formation, lipotoxicity may be developed into a metabolic therapeutic approach to suppress glioma cell growth. Future work will focus on elucidating the mechanism behind these interesting observations.

## Methods and materials

### Integrated SRS and TPF microscope for LDs imaging

**Figure 2:**
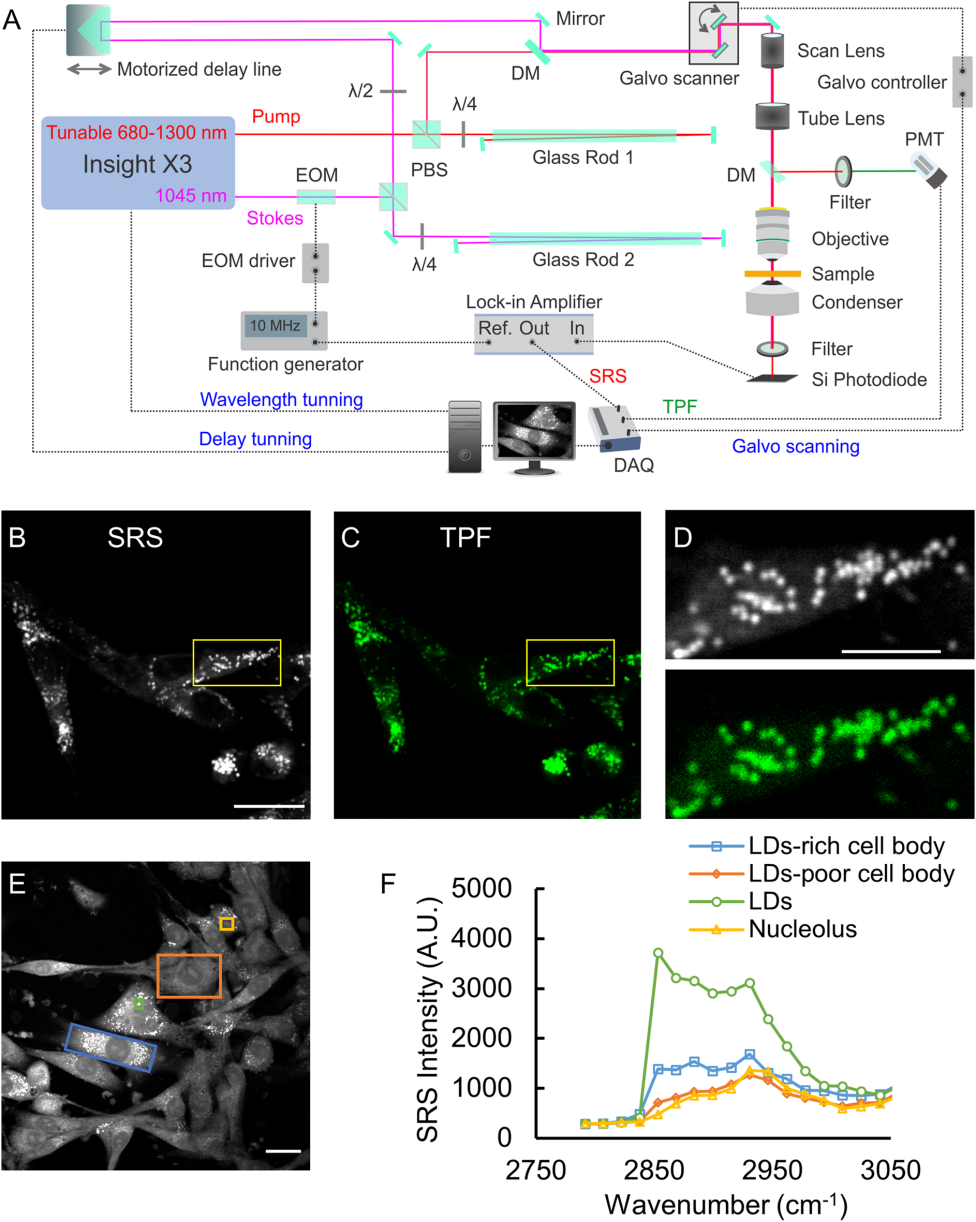
(**A**) Schematic of the optical path of the SRS microscopy system with integrated two-photon fluorescence (TPF) imaging. DM: dichroic mirror, EOM: electro-optic modulator, PBS: polarizing beam splitter, PMT: photomultiplier tube, DAQ: data acquisition card. Solid lines represent optical rays and dotted lines represent electric signals. (**B**, **C**) Paired SRS/TPF images of lipid droplets (LDs) in U87 glioma cells cultured at the regular culture condition. (**D)** Zoom-in image of (**B**, **C**) shows individual LDs. SRS image was acquired at 2854 cm^−1^. Cells in the TPF image was stained with BODIPY. (**E**) A representative SRS image of glioma cells at 2930 cm^−1^ attributed to CH_3_ vibration representing total proteins. (**F**) The spectral plots of different intracellular regions marked in the image (**E**) show distinct spectral profiles. Scale bar, 50 μm in (**B**, **C**); 20 μm in (**D**, **E**).

As shown in Fig. 2A, we built an SRS microscope based on a dual-color, tunable near-infrared laser source (Insight X3, Spectra-Physics), which provided the pump and Stokes beams for SRS imaging. A spectral focusing approach was adopted to improve spectral resolution using two SF57 glass rods^42^. The Stokes beam fixed at 1045 nm was modulated in amplitude at a radiofrequency of 10 MHz using a resonant electro-optic modulator (EOM). The tunable range of the pump beam was from 680 to 1300 nm, and therefore the system could cover all Raman shifts from 400 to 4000 cm^−1^. The pump and Stokes beams were temporarily synchronized using a motorized optical delay line and were collinearly combined to excite the sample through a water-immersion objective lens with a high numerical aperture (NA = 1.1; CFI75-Apochromat-25XC-W-1300, Nikon Inc.). The tightly focused two laser beams interacted with the sample, and during the interaction, SRS occurred. In the forward direction, the modulated Stokes beam was blocked by a short-pass optical filter (FES1000, Thorlabs Inc.) and the pump beam carrying the stimulated Raman loss (SRL) signal was measured by a large-area silicon photodiode (FDS1010, Thorlabs Inc.). The electric signal from the photodiode was fed to a lock-in amplifier (HF2LI, Zurich Instruments) to retrieve the SRL signal (i.e., the SRS signal) at the modulation frequency of 10 MHz. Laser beam scanning for imaging was realized using a two-dimensional linear galvanometer scanner (GVS002 and GPS011, Thorlabs Inc.), which was driven by the two analog outputs of a data acquisition card (DAQ, USB-6366, National Instruments). In the backward direction, a dichroic mirror (FF735-Di02-25 × 36, Semrock) was inserted in the optical path to pick up the reflected two-photon fluorescence (TPF) signal, which was then detected by a photomultiplier tube (PMT, R10699, Hamamatsu). Another optical filter (FF02-525/40-25, Semrock) was placed in front of the PMT for detection of the BODIPY fluorescence emission. The pump and Stokes beams were set to 145 mW before the objective, respectively. An integration time of 10 µs/pixel was used for laser-scanning imaging. Both the SRS and TPF signals were acquired by the same DAQ card for simultaneous imaging. The imaging system was controlled by the software ScanImage^43^.

### Cell culture

U87 glioma cells were purchased from ATCC and cultured in EMEM (ATCC) with 10% FBS (ATCC). Cells were seeded on a glass coverslip (VWR) placed in a plastic 35-mm petri dish with an estimated density of 2 × 10^5^ cells/dish for 24 h. Then cells were treated with PA, OA, or EPA, with or without 20 µM DGAT1 inhibitor T863 (Sigma-Aldrich) and 10 µM DGAT2 inhibitor PF-06424439 (Sigma-Aldrich) for additional 24 hours^40,41^. For SRS spectra acquisition and inhibition of TAG/LDs synthesis, a concentration of 100 µM was used in the FAs treatment groups. For the monitoring FAs uptake experiments, the concentrations of each FA were 0, 50, 100, and 200 µM. For the cell survival rate experiments, the concentration of OA or EPA was 200 µM, while the concentrations of PA were 200 and 400 µM. Before imaging, cells were fixed with 4% paraformaldehyde (PFA) for 10 min at room temperature and washed with PBS 3 times.

### Confocal Raman and spectral SRS imaging

Spontaneous Raman spectra of FAs were acquired using a confocal Raman spectromicroscopy (Renishaw InVia) with a 785 nm laser excitation. Aluminum foil was used as the sample substrate to minimize the fluorescence background from the glass. To acquire the spectral SRS imaging of the cells across the CH Raman band (2750–3100 cm^−1^), the wavelength of the pump beam was tuned from 812 to 789 nm. The SRS spectra of LDs were plotted from the acquired images by measuring the intensity of the pixels of the LDs.

### LDs staining with BODIPY

The fluorescent dye BODIPY (D3922, Invitrogen) was used for neutral lipid fluorescence staining. A stock solution of BODIPY with a concentration of 3.8 mM was prepared in DMSO and stored at − 20 °C. The cells were first seeded on the cover glass for 24 h and then cultured in a complete growth medium with 1.9 µM BODIPY for 10 min in the incubator. The cells were then washed with warm PBS and fixed using 4% PFA at room temperature for another 10 min. TPF imaging of LDs with BIDIPY staining was used to validate the methodology of label-free SRS imaging of LDs.

### Image processing and LDs quantification

Imaging processing was performed using Fiji (ImageJ)^44^. In this study, the ratio of the area of LDs to the area of cell bodies was used to quantify the total amount of lipids in LDs. The area of LDs was measured by thresholding the cell body background in the SRS images at 2854 cm^−1^, and the area of the cell bodies was measured by thresholding the non-cell background in the SRS images at 2854 cm^−1^. The relative unsaturation level of LDs was estimated by the SRS intensity ratio of LDs at Raman shift 3015 cm^−1^ (C=C) to 2854 cm^−1^ (CH_2_).

### Cell counting and cell survival rate

Cell counting based on white-light images was performed manually to calculate the survival rate with the number of cells. The contrast of images was enhanced using an annular filter on the white-light microscope and using simple digital enhancement. 4 digital images with a large field of view (~ 800 × 800 µm) were used for cell counting. The cell survival rate of glioma cells cultured under the regular condition was used as the control data, denoted as 100%. The cell survival rates of all experimental groups were reported in percentage relative to the regularly cultured control group. Both the mean and the standard deviation (SD) was reported in percentage.

### Statistical analysis

Linear unmixing of SRS spectra was performed using the multiple linear regression (MLR) method^45^ to quantify the composition of each FA in the LDs of glioma cells. The measured SRS spectra of the pure FAs and the SRS spectra of LDs in glioma cells cultured under the regular condition were used as the two independent variables, and the measured SRS spectra of LDs in glioma cells treated with the FAs were used as the dependent variable. All SRS spectra were background-subtracted and normalized to 1. The calculated slope coefficients were used to represent the percent composition of the free FA in the LDs. The adjusted R^2^ value was used to indicate statistical merit. For other statistical analyses, the independent two-sample t-test method was performed to obtain the p-values in Microsoft Excel.

## Results

### Photostable imaging of LDs with SRS

SRS at 2854 cm^−1^ corresponding to the CH_2_ chemical bond vibration was used to image label-free lipid droplets (LDs) in fixed U87 cells. To validate the specificity and selectivity of SRS imaging of LDs, we stained LDs in glioma cells using the fluorescent dye BODIPY and performed simultaneous SRS/TPF imaging of the same sample. As shown in Fig. 2B,C, images of LDs with SRS and TPF looked identical. As shown in Fig. 2D, co-localization of single LDs was achieved between SRS and TPF imaging, and tiny LDs with a dimension down to 1 μm were clearly visualized in SRS images using the high NA objective lens. We also found that label-free SRS imaging of LDs exhibited higher photostability than BODIPY-stained fluorescence imaging. The reliability of imaging label-free LDs with SRS has been validated comparing with traditional methods in prior studies^30,46,47^.

SRS spectral imaging was obtained frame-by-frame while tuning the wavelength of the pump beam (i.e., the Raman shift) and adjusting the optical delay line accordingly (Fig. 2A). Figure 2F shows the SRS spectra of different cellular regions marked in Fig. 2E, which was a representative image at 2930 cm^−1^ (CH_3_ vibration) from the acquired spectral imaging stack. From the SRS spectra data, we find that different cellular areas have distinct spectral profiles, demonstrating the feasibility of using SRS spectral imaging to analyze the chemical composition of biological samples.

### Tracking FAs encoding into lipids of LDs with SRS spectral imaging

**Figure 3:**
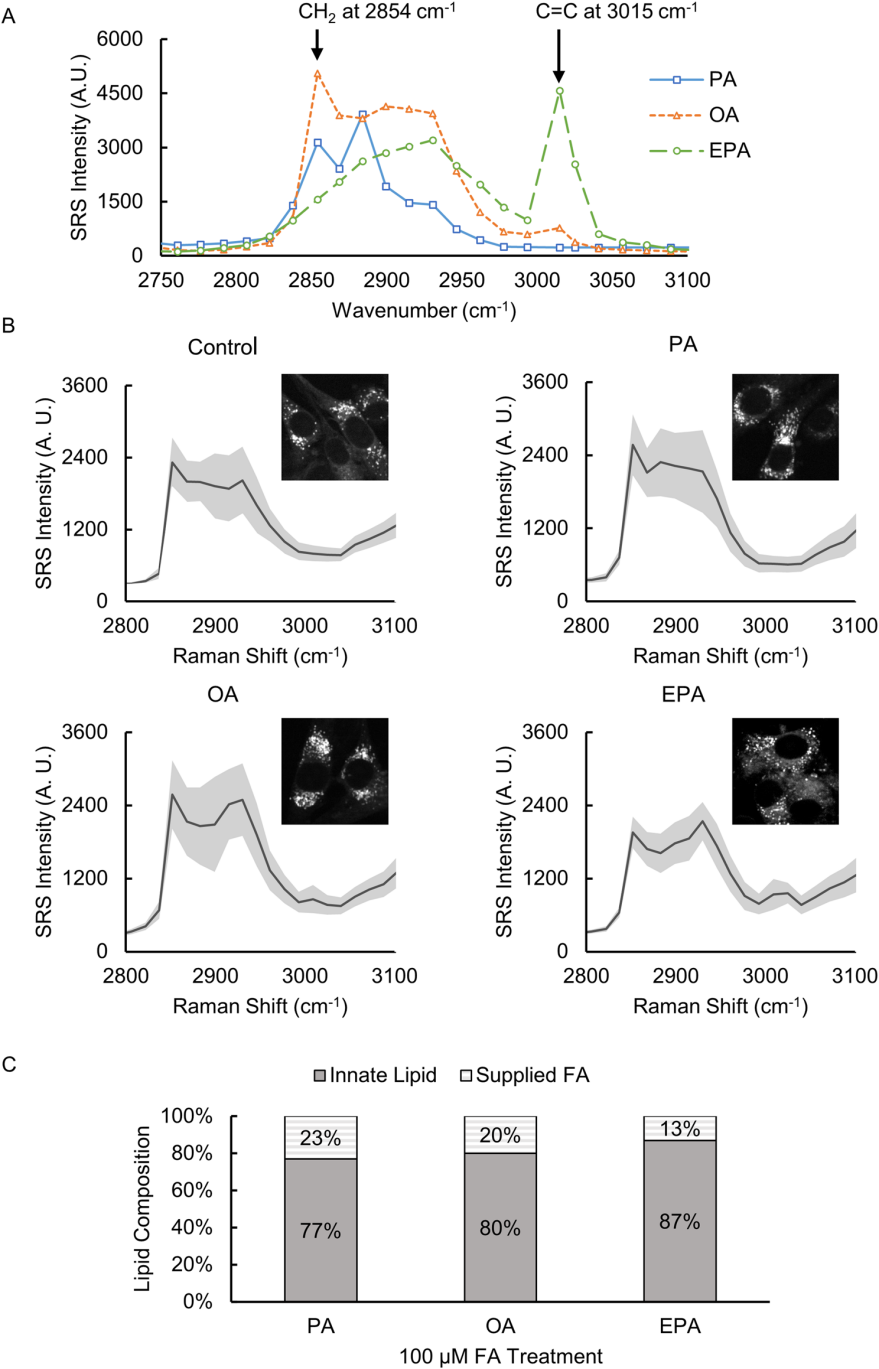
(**A**) SRS spectra of fatty acids PA, OA, and EPA. (**B**) Average SRS spectra of all LDs with diameters above 1 µm from SRS images of U87 glioma cells treated with PA, OA, and EPA at 100 μM for 24 h, respectively. The solid line shows the mean SRS intensity, and the gray area shows the standard deviation (SD). (**C**) The percent compositions of FAs in the LDs calculated through linear unmixing of the SRS spectra using the multiple linear regression (MLR) method. The adjusted R^2^ was 0.972, 0.976, and 0.954, for PA-, OA- and EPA-treated LDs, indicating this was a well-fitting regression.

We used the established SRS spectral imaging approach to analyze the lipid composition of the LDs. We first examined the SRS spectra in the high wavenumber region (2750–3100 cm^−1^) of the three FAs (PA, OA, and EPA) that we used to treat the glioma cells in this study. We confirmed that the SRS spectra of FAs (Fig. 3A) were nearly identical to the Raman spectra measured using a commercial Raman spectroscopy (Fig. S1). From Fig. 3A, we see that the three FAs have distinct spectral features. At 2854 cm^−1^, PA and OA show a clear peak attributed to the CH_2_ vibration, but EPA does not show a clear peak at this Raman shift. In this study, we used SRS imaging at 2854 cm^−1^ to represent the total lipids for visualization of LDs. In addition, SRS imaging at 3015 cm^−1^ attributed to the C=C double bond vibration was used to evaluate the relative unsaturation level of lipids in the LDs^48^. It is clear that EPA shows the strongest signal at 3015 cm^−1^ because each EPA molecule contains five C=C double bonds (Fig. S1A).

We treated U87 glioma cells with 100 µM PA, OA, and EPA, respectively, for the SRS spectral imaging. As shown in Fig. 3B, we measured all LDs with diameters above 1 μm in around 40 glioma cells and plotted their SRS spectra with the mean and the standard deviation (SD). We observed that the SRS spectra of LDs (Fig. 3B) exhibited the characteristic Raman spectral features of the FA (Fig. 3A) that was used to treat the cells in each experimental group, indicating that the supplied free FAs were largely incorporated into the neutral lipids stored in LDs. To quantitatively estimate the percent composition of each FA in LDs, we performed spectral linear unmixing using the multiple linear regression (MLR) method^45^. The data showed that FA-treated LDs contained ~ 23% of PA, ~ 20% of OA, and ~ 13% of EPA, respectively, relative to the innate LDs in the untreated glioma cells (Fig. 3C). This result demonstrates that using the SRS spectral imaging approach, the “flow” of FAs into LDs can be tracked and quantified in a label-free manner. In addition, the large SD values indicate that the chemical compositions of the LDs were heterogeneous even in cells treated with pure FA. This observation was consistent with the previous studies^47,49–51^.

### Monitoring LDs formation with two-color SRS imaging

**Figure 4:**
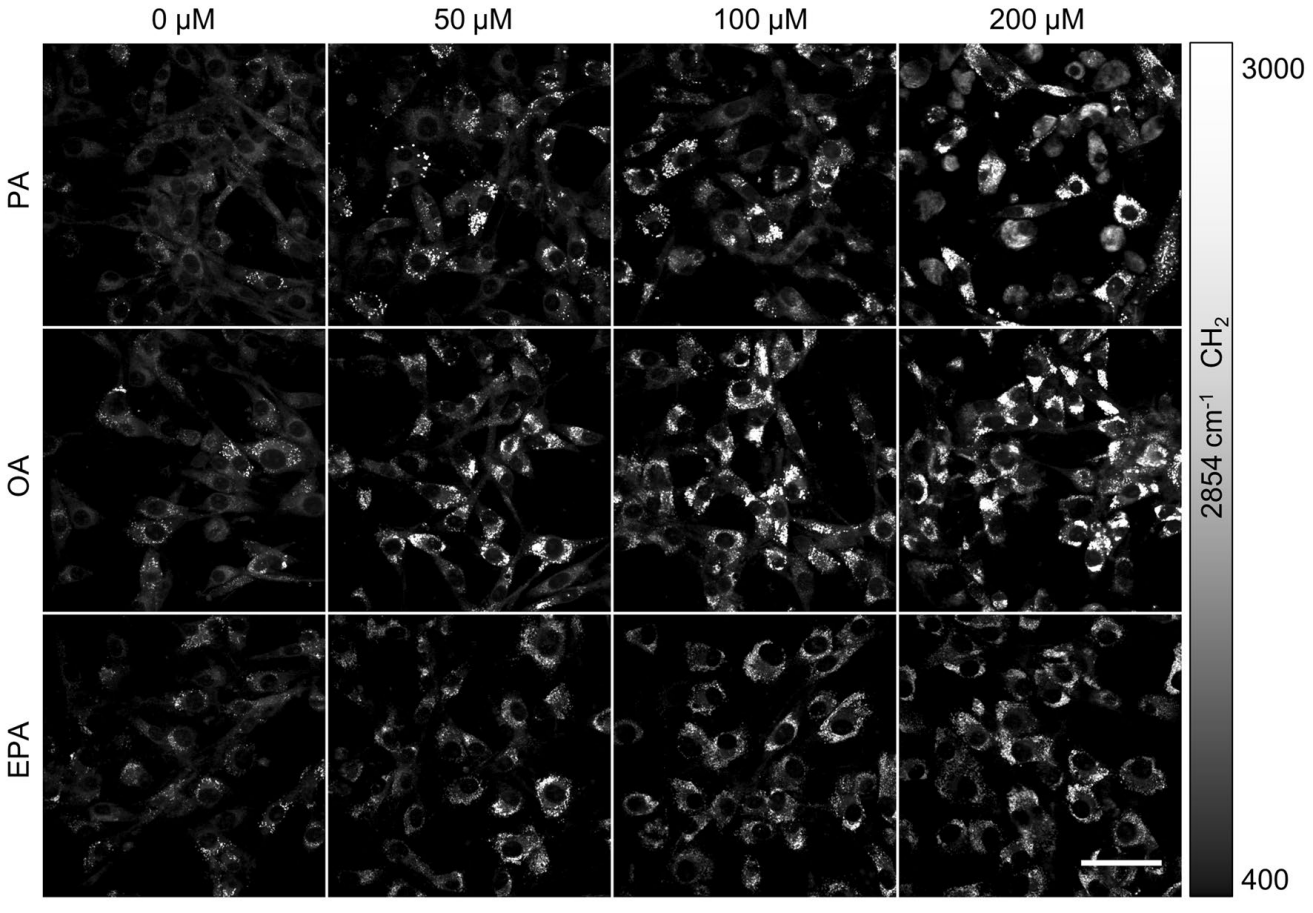
Representative SRS images of LDs at 2854 cm^−1^ in U87 glioma cells treated with free FAs: PA, OA, and EPA, respectively, at different concentrations (0, 50, 100, 200 μM) for 24 h. Supplies of free FAs in the culture medium increased LDs synthesis in U87 glioma cells. Scale bar, 50 μm.

**Figure 5:**
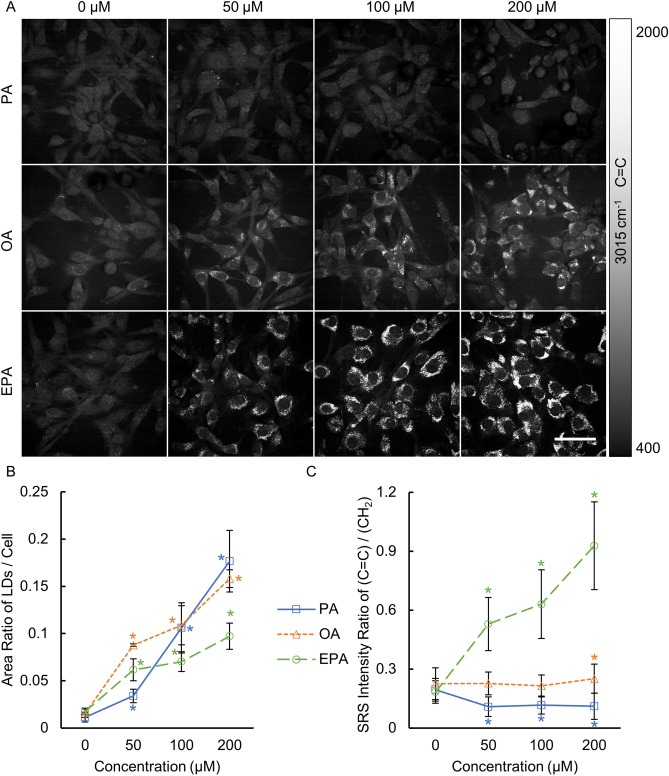
(**A**) SRS images at 3015 cm^−1^ attributed to C=C vibration with the corresponding fields of view as in Fig. 4. U87 glioma cells were treated with PA, OA, and EPA, respectively, at different concentrations (0, 50, 100, 200 μM) for 24 h. Scale bar, 50 μm. (**B**) Quantification of total lipids in the LDs using the area-ratio method with SRS images at 2854 cm^−1^. (**C**) Quantification of the relative unsaturation level of lipids in LDs with SRS images at 2854 cm^−1^ and 3015 cm^−1^. *P < 0.05, the difference is statistically significant from 0 µM (the untreated control group) for all PA-, OA-, and EPA-treated groups.

We then treated U87 glioma cells with the three free FAs: PA, OA, and EPA, respectively, at different concentrations 0, 50, 100, 200 µM for 24 h. We imaged the cells at 2854 cm^−1^ (CH_2_, Fig. 4) and 3015 cm^−1^ (C=C, Fig. 5A) with SRS. As shown in Fig. 4, we observed that all three FAs induced extra LDs deposition in glioma cells, and the higher dose of the free FAs, the more LDs were generated. It is noted that because of the different SRS signal levels at 2854 cm^−1^ of the three FAs (Fig. 3A), quantitative comparison of LDs may not be performed solely based on the lipid signal intensity. Instead of using the signal intensity, we used the area of LDs relative to the area of the cell bodies for the quantification of LDs (Fig. 5B). Our area-based quantification showed that 200 µM PA and OA treatments increased LDs by 15-fold and tenfold, respectively. However, 200 µM EPA treatment increased LDs by only a fivefold, indicating that U87 glioma cells uptake EPA at a lower efficiency than PA or OA. This observation was also confirmed by the result shown in Fig. 3C. In general, all three FAs increased the LDs accumulation in U87 cells in a dose-dependent manner.

The SRS signal intensity ratio of LDs at Raman shift 3015 cm^−1^ (C=C) to 2854 cm^−1^ (CH_2_) was used to estimate the relative unsaturation level of LDs in glioma cells (Fig. 5C). It is not surprising that EPA strongly increased the unsaturation level of the lipids in LDs (i.e., 2.9-, 3.4-, and 5.1-fold at 50, 100, 200 μM, respectively, compared to the untreated LDs) because EPA is a polyunsaturated fatty acid. We also notice that while OA treatment maintained nearly the same unsaturation level of lipids as the untreated cells, PA treatment decreased the unsaturation level of lipids in LDs by ~ 40% to 50%. Note that cells can maintain membrane fluidity as well as other functions by regulating the lipid saturation degree^52^. Collectively, our results reveal that a high-dose supply of FAs not only dramatically increased LDs deposition in glioma cells, but also strongly affected the chemical composition and saturation level of LDs, which would therefore influence cell functions.

### Inhibition of DGAT1/2 depleted LDs in glioma cells

**Figure 6:**
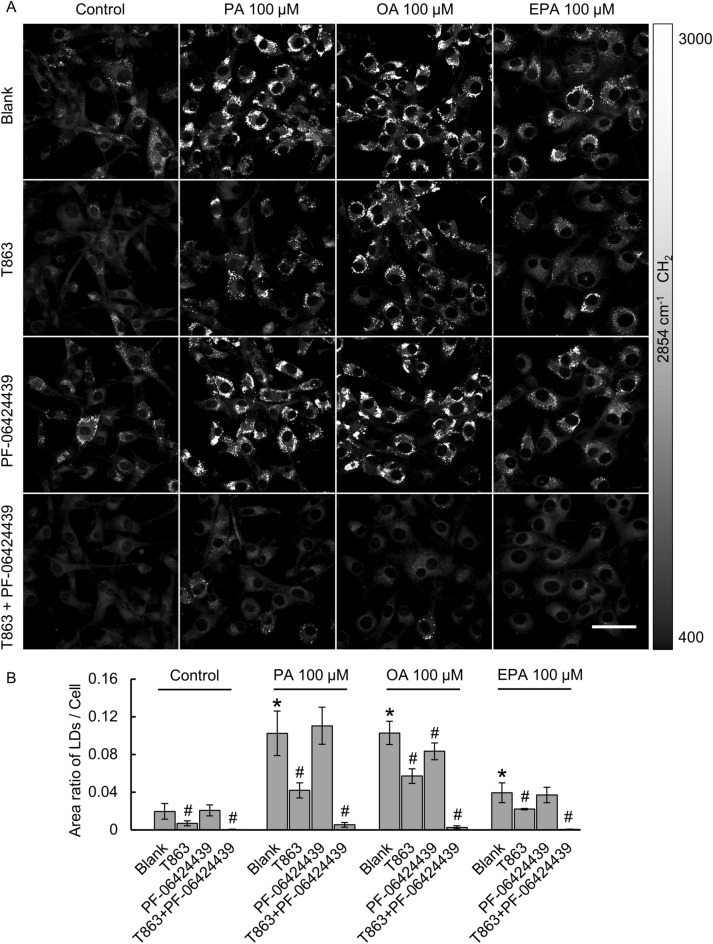
(**A**) SRS imaging of LDs at 2854 cm^−1^ under various culture conditions. U87 glioma cells were treated with PA, OA, and EPA at 100 μM for 24 h. DGAT1 and DGAT2 are essential enzymes for LDs synthesis in U87 cells. DGAT1/2 were inhibited by T863 (20 µM) and PF-06424439 (10 µM). Scale bar, 50 μm. (**B**) Quantification of total LDs using the area-ratio method for each experimental group with SRS images at 2854 cm^−1^. *P < 0.05, the difference is statistically significant from blank in the control group. ^#^P < 0.05, the difference is statistically significant from blank in control for all PA-, OA-, and EPA-treated groups.

DGAT1/2 are essential enzymes to assemble FAs onto a glycerol backbone for TAG biosynthesis^38^. As illustrated in Fig. 1C, we used two selective inhibitors T863 and PF-06424439 to block DGAT1 and DGAT2, respectively^40,41^. U87 glioma cells were cultured in a growth medium with 100 µM PA, 100 µM OA, or 100 µM EPA with or without 20 μM T863 and 10 μM PF-06424439. The imaging results are shown in Fig. 6A and quantification of LDs is shown in Fig. 6B. Under all conditions with or without FAs treatment, we found that blocking DGAT1 reduced LDs formation more efficiently than blocking DGAT2, indicating that DGAT1 may play a more essential role than DGAT2 in U87 cells. Under the regular culture condition or with the supply of the three FAs at 100 µM, blocking either DGAT1 or DGAT2 alone could not stop LDs formation. However, we found that blocking both DGAT1 and DGAT2 completely depleted LDs formation under all culture conditions.

### Depletion of PA-treated LDs rescued U87 glioma cells

**Figure 7:**
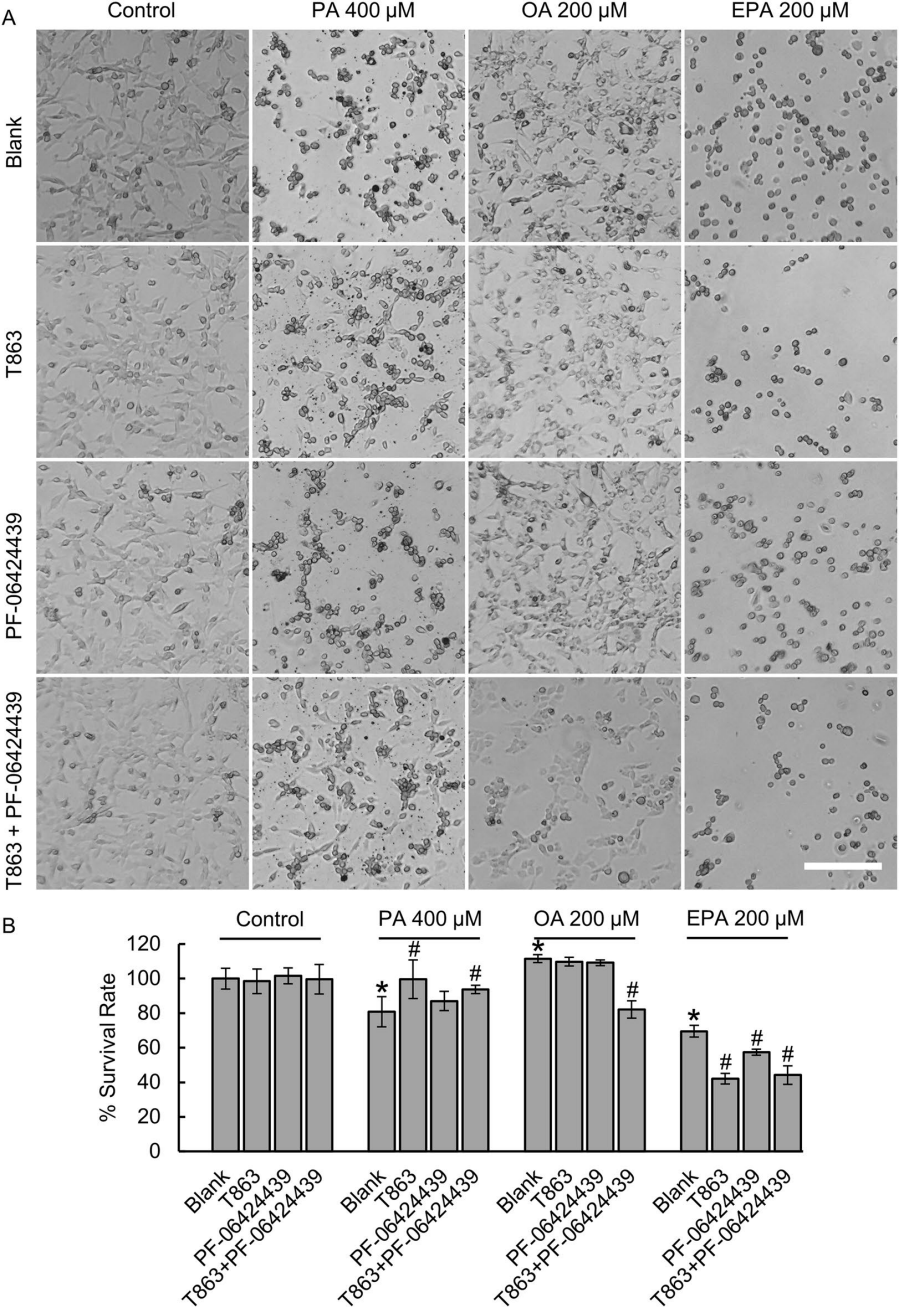
(**A**) Representative images collected using a white-light microscope of U87 glioma cells treated with 400 µM PA, 200 µM OA, or 200 µM EPA, with or without 20 µM T863 or 10 µM PF-06424439 treatments. LDs protected U87 glioma cells in OA- and EPA- but not PA-induced lipotoxicity. Scale bar, 150 µm. (**B**) Quantification of the survival rates of glioma cells under various culture conditions in (**A**). *P < 0.05, the difference is statistically significant from blank in the control group. ^#^P < 0.05, the difference is statistically significant from blank in control for all PA-, OA-, and EPA-treated groups.

We performed cell counting with white-light microscopic images (Fig. 7A) to evaluate the cell survival rates under various treatment conditions with FAs and/or DGAT1/2 inhibitors (Fig. 7B). The cells cultured under a regular condition served as the control group, whose survival rate was denoted as 100%. The survival rates of treated groups were reported relative to the untreated control group (see “Methods and materials”). We found that 200 µM PA treatment did not induce measurable changes in the cell survival rate (Fig. S2). We then increased the PA concentration to 400 µM and observed a moderate reduction in cell survival rate (mean: 80.8%, SD: ± 8.7%). However, after we depleted LDs formation in the 400 µM PA-treated cells, we were able to rescue the cell growth (99.7% ± 1.2% for T863 only, and 93.8% ± 2.3% for T863 and PF-06424439 combined treatment) (Fig. 7B). These results indicate that inhibition of the synthesis of PA into LDs was beneficial for glioma cell growth.

### Depletion of OA- and EPA-treated LDs reduced cell survival rate

For 200 µM OA-treated cells, we observed a small increment (111.6% ± 2.2%) of the cell proliferation rate. But when LDs were completely depleted in the OA-treated cells using the two inhibitors, cell survival rate dropped (82.2% ± 5.1%) (Fig. 7B). These results indicate that uptake of OA was beneficial for glioma proliferation and it may be inadvisable to introduce lipotoxicity by treating extra OA on purpose^23,53^. Most interestingly, 200 µM EPA-treated cells underwent a clear drop in cell survival rate (69.6% ± 3.5%) showing its highest lipotoxicity to U87 glioma cells. When LDs were depleted in EPA-treated cells, lipotoxicity was further intensified, which was evidenced by the significant drop of the cell survival rate to 44.2% ± 5.44% (Fig. 7B). From these results we summarize that depending on the FA types, LDs may play opposed roles in managing lipotoxicity. Among the three FAs that we tested, EPA shows the highest lipotoxicity to U87 glioma cells.

Previous studies indicate that LDs formation may be a protective mechanism for cells to manage stresses such as lipotoxicity imposed by free FAs at a high concentration^12,53^. This stress may be the molecular driving force for cells to efficiently convert free FAs into neutral lipids, which will then be stored in LDs. When TAG synthesis and LDs formation are inhibited, the concentration of free FAs and other lipid species in the cytosol will be largely elevated, imposing higher lipotoxicity to the cells^11,54^. However, our experimental results reveal that the relationship between lipotoxicity and LDs may be more complicated than what has been discovered before.

## Discussion

In this study, we sought to evaluate the possibility of using lipotoxicity to suppress GBM growth. We observed that glioma cells produced more LDs upon free FAs treatment. SRS spectral imaging was used to trace the flow of supplied free FAs into TAGs and then into LDs. This may be because glioma cells have the ability to reprogram their lipid metabolism pattern based on their growth environment, which grants glioma cells high tolerance to lipotoxicity via LDs formation. To assess the role of LDs in lipotoxicity, we managed to inhibit the final step of TAG biosynthesis and LDs formation and we conclude that LDs may or may not protect against lipotoxicity depending on the types of FAs. More specifically, inhibition of PA-induced LDs unexpectedly rescued U87 cells. However, depletion of OA- and EPA-induced LDs decreased the cell survival rate.

We tested one saturated fatty acid (PA), one monounsaturated fatty acid (OA), and one polyunsaturated fatty acid (EPA) to assess lipotoxicity in U87 glioma cells. All of them showed a clear increment of LDs dose-dependently, and lipotoxicity was observed with PA and EPA treatments. We then inhibited the synthesis of TAG by blocking the DGAT1 and DGAT2. The survival rate of OA and EPA treated cells was decreased in the absence of LDs, indicating that LDs protect glioma cells from unsaturated FAs-induced lipotoxicity. But inhibition of LDs rescued PA-induced lipotoxicity, the reason for which is unclear. It has been reported that PA or EPA treatment-induced apoptosis while OA treatment increased the proliferation rate of glioma cells^23,36,37^, which are consistent with our results. And the inhibition of DGAT1 in PA-treated cells would increase C16 ceramide which induced tumor growth while C18 ceramide did not^11,55^, inferring that inhibition of PA-induced LDs may lead to a worse outcome in treating gliomas. Therefore, inhibition of LDs for potential GBM treatment must consider the composition of FAs. Other lipid species-induced tumor growth could still dominate even in the presence of lipotoxicity. The inhibition of LDs may cause additional issues that are beyond the scope of the current study.

DGAT1 and DGAT2 are both essential in TAG synthesis^38,40,41^. DGAT1 inhibitor could suppress the formation of ~ 50% LDs in U87 cells. And over 90% inhibition could be achieved together with DGAT2 inhibited. But PF-06424439 alone seemed no clear impact in regular culture condition, PA, or EPA treatment. Since DGAT2 is related to LDs expansion, the inhibition of DGAT2 could be a reason for reduced OA-induced LDs synthesis with PF-06424439, yet the impact of DGAT2 in other FAs treatments may be less sensitive than in OA.

It’s reported that DGAT1 protects cells from lipid-induced ER stresses by OA re-esterification during lipolysis^54^. DGAT1 inhibition increased free OA releasing into the culture environment while additional DGAT2 inhibition was similar to DGAT1 inhibition alone. The broader substrates of DGAT1 may cause more severe FA accumulation than DGAT2 during inhibition^56^. In our case, we found clear lipotoxicity in OA and EPA treatments caused by DGAT1 and DGAT2 inhibition (Fig. 7), which may be explained by the inhibition of FAs re-esterification. We also noticed that DGAT2 played an important role to assist cell protection from OA-induced lipotoxicity while inhibition of DGAT1 alone did not affect the cell viability with OA treatment. Therefore, both DGAT1 and DGAT2 are involved in lipotoxicity in GBM. But the conclusion varies for different FA types that DGAT1/2 inhibition in OA and EPA groups induced ER stress while DGAT1/2 inhibition in PA treatment increased cell proliferation instead of inducing lipotoxicity.

The different levels of unsaturation of FAs led to various glioma reaction^37^. However, it is technically laborious to measure the unsaturation degree of lipids in cells using traditional methods. Label-free SRS microscopy is a powerful tool in lipid imaging with rapid speed and high spatial resolution. The major chemical composition and relative unsaturation level of LDs could also be analyzed with SRS spectral imaging data. In addition, recent studies reveal that non-polar lipids may also aggregate outside of the LDs in cells^39,57^. In our study, we did not capture non-spherical lipid-rich aggregates that had a large dimension above 1 μm. Due to the diffraction-limited spatial resolution of SRS, lipid aggregates or LDs that were below 1 μm were not quantified in our analysis. Nevertheless, Raman spectroscopy has been used to analyze the polarity of lipids^58^.

As demonstrated in this study, one advantage of SRS imaging is its high imaging speed with amplified signals compared with spontaneous Raman imaging. It is noted that full spectral data collection is more technically difficult with SRS than with Raman^27,48^. Compared to fluorescent imaging, SRS can be label-free, which avoids tedious sample staining procedures. However, fluorescent imaging can readily achieve single-molecule sensitivity while SRS imaging sensitivity is largely limited by the small Raman cross-section and the cellular or tissue background^29,59^. SRS can be used to image the native LDs in fixed or live cells and tissue, but high-throughput lipid profiling still has to use other technologies such as chromatography or mass spectrometry^60^.

In summary, SRS microscopy was used to study lipid metabolism in U87 glioma cells upon fatty acids treatments. In this study, we demonstrate that lipotoxicity and abnormal accumulation of lipid droplets could be a potential therapeutic target to kill glioma cells. We found that 400 µM PA and 200 µM EPA treatments decreased U87 cell viability. We also illustrated that lipid droplets may play opposed roles in managing lipotoxicity. Based on the results presented here, we summarize that free EPA treatment with depletion of lipid droplets may be used as a potential therapeutic approach to suppress the growth of glioma cells. Future work will focus on understanding the effect and mechanism of why EPA imposes the highest lipotoxicity in glioma cells using different cell lines or animal models.

## Supplementary Information

Supplementary Information.

## Data Availability

The datasets generated and analyzed during the current study are available from the corresponding author on reasonable request.
