# Supplementary material for: Assessing fatty acid-induced lipotoxicity and its therapeutic potential in glioblastoma using stimulated Raman microscopy
Source: Sci Rep. 2021 Apr 1;11:7422. doi: 10.1038/s41598-021-86789-9 (PMC8016949; doi:10.1038/s41598-021-86789-9)
Supplement: Supplementary file 1 — Supplementary Information. [file 41598_2021_86789_MOESM1_ESM.pdf]

Assessing fatty acid-induced lipotoxicity and its therapeutic potential in glioblastoma using stimulated Raman microscopy

Yuhao Yuan[1], Niraj Shah[2], Mohammad I. Almohaisin[1], Soumit Saha[1], and Fake Lu[1]*

[1]Binghamton University, State University of New York, Department of Biomedical Engineering, Binghamton, NY 13902, USA

[2]Binghamton University, State University of New York, Department of Psychology, Binghamton, NY 13902, USA

*To whom correspondence should be addressed. Email: fakelu@binghamton.edu

Supplementary Figures

Figure S1

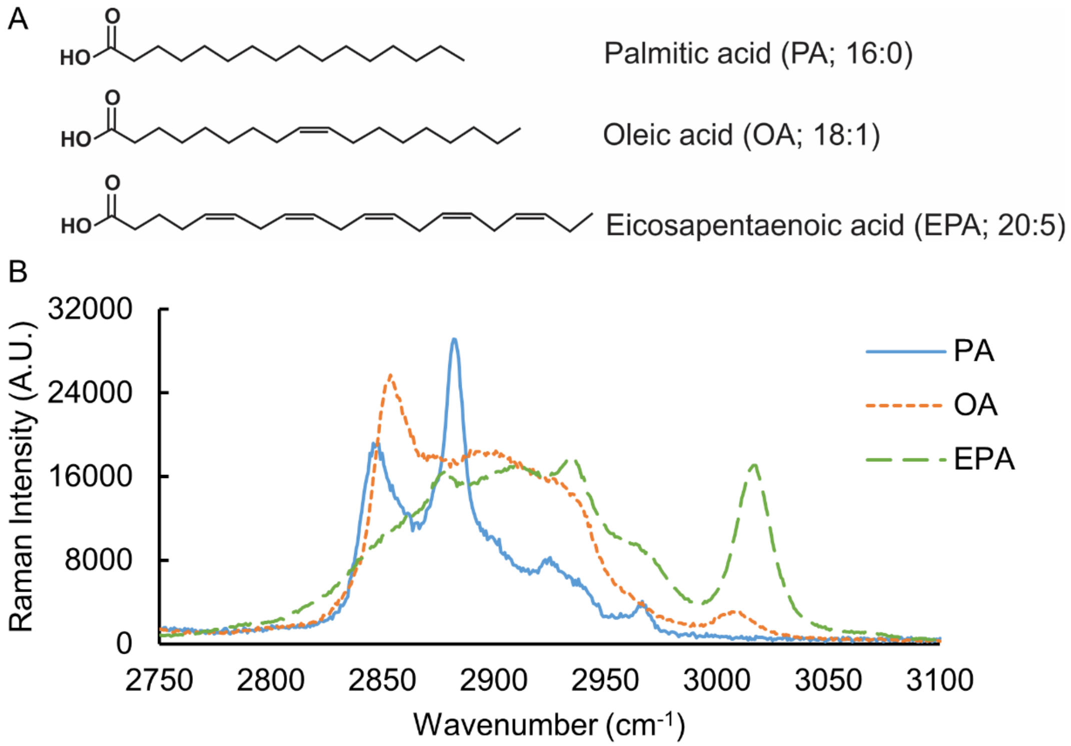

Figure S1: **(A)** The molecular structure of PA, OA, and EPA. **(B)** Raman spectra of PA, OA, and EPA acquired using a confocal Raman spectroscopy (Renishaw InVia) were nearly identical to the SRS spectra acquired using the lab-built SRS microscope. Supplementary data to Fig. 3.

Figure S2

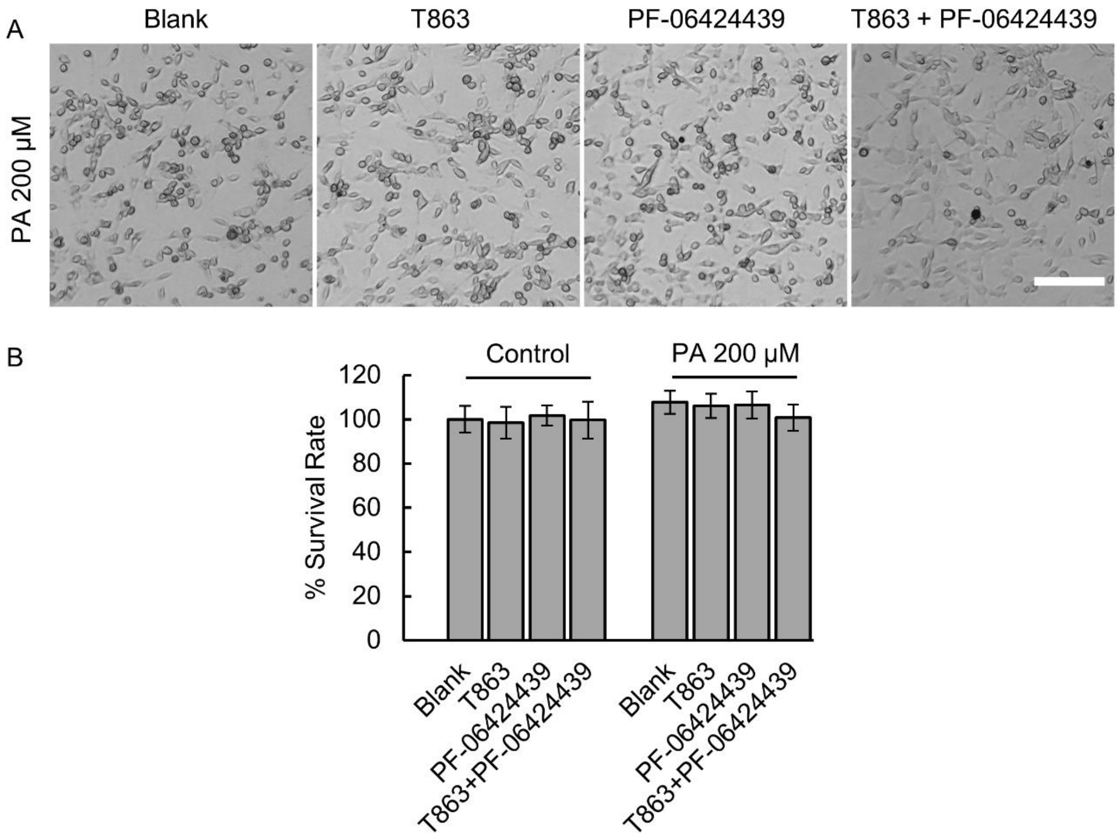

Figure S2. **(A)** Representative white-light microscopic images of U87 glioma cells treated with 200 µM PA, with or without inhibitors 20 µM T863 or 10 µM PF-06424439 treatment. Scale bar, 150 µm. **(B)** Plots of survival rates quantified by cell counting of 4 images in (A) for each group, which shows that 200 µM PA treatment did not reduce cell growth compared with the control group. Supplementary data to Fig. 7.
